# Characterization of Discrete Phosphopantetheinyl Transferases in *Streptomyces tsukubaensis* L19 Unveils a Complicate Phosphopantetheinylation Network

**DOI:** 10.1038/srep24255

**Published:** 2016-04-07

**Authors:** Yue-Yue Wang, Xiao-Sheng Zhang, Hong-Dou Luo, Ni-Ni Ren, Xin-Hang Jiang, Hui Jiang, Yong-Quan Li

**Affiliations:** 1 College of Life Sciences, Zhejiang University, Hangzhou, 310058, China; 2 College of Pharmaceutical Sciences, Zhejiang University, Hangzhou, 310058, China; 3 Zhejiang Provincial Key Laboratory for Microbial Biochemistry and Metabolism Engineering, Hangzhou, 310058, China

## Abstract

Phosphopantetheinyl transferases (PPTases) play essential roles in both primary metabolisms and secondary metabolisms via post-translational modification of acyl carrier proteins (ACPs) and peptidyl carrier proteins (PCPs). In this study, an industrial FK506 producing strain *Streptomyces tsukubaensis* L19, together with *Streptomyces avermitilis*, was identified to contain the highest number (five) of discrete PPTases known among any species thus far examined. Characterization of the five PPTases in *S. tsukubaensis* L19 unveiled that *stw* ACP, an ACP in a type II PKS, was phosphopantetheinylated by three PPTases FKPPT1, FKPPT3, and FKACPS; *sts* FAS ACP, the ACP in fatty acid synthase (FAS), was phosphopantetheinylated by three PPTases FKPPT2, FKPPT3, and FKACPS; TcsA-ACP, an ACP involved in FK506 biosynthesis, was phosphopantetheinylated by two PPTases FKPPT3 and FKACPS; FkbP-PCP, an PCP involved in FK506 biosynthesis, was phosphopantetheinylated by all of these five PPTases FKPPT1-4 and FKACPS. Our results here indicate that the functions of these PPTases complement each other for ACPs/PCPs substrates, suggesting a complicate phosphopantetheinylation network in *S. tsukubaensis* L19. Engineering of these PPTases in *S. tsukubaensis* L19 resulted in a mutant strain that can improve FK506 production.

Phosphopantetheinyl transferases (PPTases) catalyze the phosphopantetheinylation of acyl carrier proteins (ACPs) in polyketide synthases (PKSs), peptidyl carrier proteins (PCPs) in nonribosomal peptide synthetases (NRPSs), and ACPs in fatty acid synthases (FASs) from inactive apo-forms into active holo-forms^1^,^2^,^3^. Thus, PPTases are essential to both primary metabolisms and secondary metabolisms in various species. PPTases can be classified into three groups according to their structures. A group I PPTase, also named as an ACPS-type PPTase, consists of three identical peptide subunits with about 120 amino acid residues in each subunit^4^,^5^,^6^. A group II PPTase, also named as a Sfp-type PPTase, consists of one peptide which is about twice the size of one group I PPTase subunit^7^,^8^,^9^. A group III PPTase exists as a domain of a FAS or a PKS^10^,^11^.

A group III PPTase prefers to phosphopantetheinylate the ACP, which locates with it in the same peptide^10^,^11^. A discrete PPTase, a group I PPTase or a group II PPTase, should phosphopantetheinylate multiple ACPs/PCPs, since the number of PPTases is much less than that of ACPs/PCPs in all known species 12,13,14,15. Animals and Plants usually contain one to three group II PPTases^16^. Few bacteria harbor only one group II PPTase which phosphopantetheinylate ACPs/PCPs involved in both primary metabolism and secondary metabolism, such as *Pseudomonas aeruginosa, Nodularia spumigena* NSOR10, *Synechocystis* sp. PCC6803, and *Haemophilus influenza*^17^,^18^,^19^,^20^. Most bacteria have one group I PPTase and no less than one group II PPTases^12^,^13^,^14^,^15^. In *Streptomyces coelicolor* and *Streptomyces chattanoogensis* L10, it has been reported that group I PPTases prefer to phosphopantetheinylate ACPs in type II FASs and type II PKSs, while group II PPTases prefer to phosphopantetheinylate ACPs in type I PKSs^12^,^13^. Recently, antibiotic production has been improved by engineering of PPTases^13^.

FK506 (tacrolimus) is a clinical immunosuppressant widely used after allogeneic kidney, liver, and heart transplantations^21^,^22^,^23^,^24^. Industrial production of FK506 is made by fermentation of some *Streptomyces* strains. Although FK506 is known to be biosynthesized by a PKS/NRPS hybrid and partial biosynthetic pathway of FK506 has been elucidated^25^,^26^,^27^,^28^, phosphopantetheinylation of ACPs/PCPs in FK506 biosynthetic PKS/NRPS has never been studied to date. An FK506 producing strain, *Streptomyces tsukubaensis* L19 was isolated from Yunnan, China by our group and was used for industrial production of FK506. In this study, we identified one group I PPTase and four group II PPTases in an industrial FK506 producing strain *S. tsukubaensis* L19. Characterization of these PPTases unveiled that the functions of these PPTases complement each other, suggesting a complicate phosphopantetheinylation network in *S. tsukubaensis* L19. A mutant strain with higher FK506 production and shorter fermentation time was also constructed by engineering of these PPTases.

## Results and Discussion

### Analysis of discrete PPTase genes and PKS gene clusters in *Streptomyces tsukubaensis* L19

**Figure 1:**
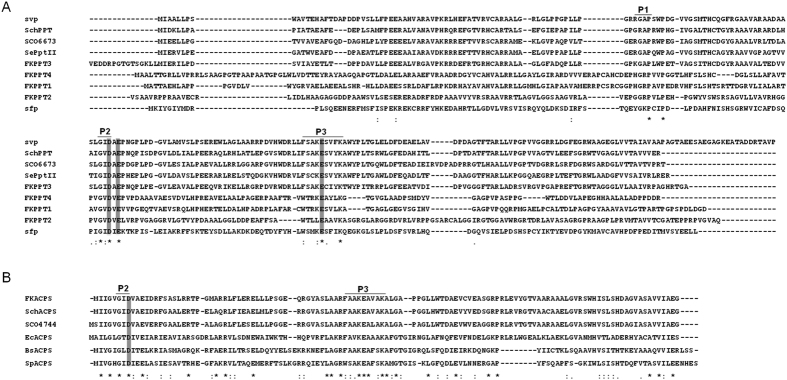
Sequence alignment of group II PPTases (**A**) and group I PPTases (**B**). Conserved motifs P1, P2, and P3 are indicated. The proposed magnesium binding residues are shaded. svp (GenBank accession Nos AAG43513), SchPPT (JQ283111), SCO6673 (NP_630748), SePptII (A4FC68), sfp (BAA09125), SchACPS (JQ283112), SCO4744 (OO86785), EcACPS (P24224), BsACPS (CAB12269), SpACPS (AAG22706).

The whole genomic DNA of *S. tsukubaensis* L19 was recently sequenced to contain more than 7.9 M base pairs and 7,000 open reading frames (ORFs). Analysis of the genomic sequence revealed five discrete PPTase genes, *FKPPT1, FKPPT2, FKPPT3, FKPPT4*, and *FKACPS* (GenBank accession numbers KT582112-KT582116). Neither of these genes is clustered with any secondary metabolite biosynthesis gene clusters. Alignment of these PPTases with known PPTases showed that FKPPT1, FKPPT2, FKPPT3, FKPPT4 contains three conserved motifs, PRWP, GID and FSAKESVYK, found in the Sfp-type PPTase motifs P1, P2 and P3, while FKACPS contains just last two conserved motifs found in ACPS-type PPTase. Thus, FKPPT1, FKPPT2, FKPPT3, and FKPPT4 belong to the Sfp-type PPTase group, and FKACPS belongs to the ACPS-type PPTase group, respectively (Fig. 1).

Most bacteria contain two to three discrete PPTases, such as *E. coli, S. coelicolor, S. chattanoogensis* L10^12^,^13^. To date, *Streptomyces avermitilis* is the known to harbor the highest number (five) of discrete PPTases^1^,^29^. Together with *S. avermitilis, S. tsukubaensis* L19 contains the highest number of discrete PPTases known among any species thus far examined.

**Figure 2:**
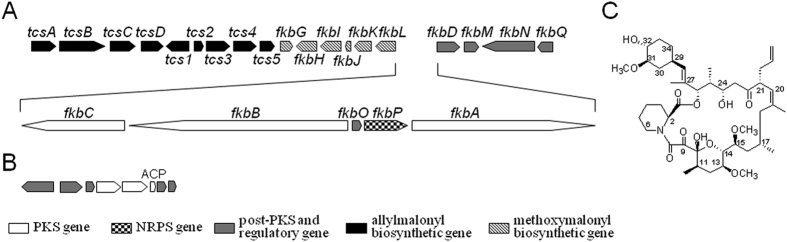
FK506 biosynthetic gene cluster (**A**), spore pigment biosynthetic gene cluster (**B**) in *S. tsukubaensis* L19, and the molecular structure of FK506 (**C**).

Analysis of the genomic sequence of *S. tsukubaensis* L19 revealed about thirty proposed PKS, NRPS, and PKS-NRPS hybrid gene clusters. The FK506 biosynthetic gene cluster in *S. tsukubaensis* L19 showed 100% DNA identity with that in *Streptomyces tsukubaensis* YN06^27^,^28^. A type II PKS was named as *stw* PKS (*Streptomyces tsukubaensis whiE*), since its gene cluster contains homologous genes in *whiE* PKS gene clusters, which are involved in spore pigment biosynthesis in several *Streptomyces* strains (Fig. 2)^30^,^31^,^32^. More than one hundred proposed ACPs and PCPs within these PKSs, NRPSs, and PKS-NRPS hybrids were potential substrates of these five PPTases.

### *In vitro* phosphopantetheinylation system

To characterize if these five PPTases have activities, an *in vitro* co-expression system was built up. TcsA-ACP (the ACP domain in the allylmalonyl unit biosynthetic module in FK506 biosynthetic PKS/NRPS), FkbP-PCP (the PCP domain in the NRPS module in FK506 biosynthetic PKS/NRPS), *stw* ACP (the ACP in *stw* PKS), and *sts* FAS ACP (the ACP in *S. tsukubaensis* L19 FAS) were selected as substrates of PPTases. First, *tcsA-ACP, fkbP-PCP* (fused with *SUMO* gene), *stw ACP*, and *sts FAS ACP* were individually cloned into pET28a, resulting in four *ACP/PCP*-containing-plasmids pET-AACP, pYY0081, pYY0082, and pYY0098, respectively. Second, *FKPPT1, FKPPT2, FKPPT3, FKPPT4*, and *FKACPS* were individually cloned into the *Nde*I/*Hin*dIII sites of pYY0040^16^, in which both His-tag gene and Nus-tag genes were deleted from pET44a, resulting in five *PPTase*-containing-plasmids, pYY0072, pYY0078, pYY0073, pYY0077, and pYY0074, respectively. Finally, four *E. coli* BL21(DE3) harboring both an *ACP/PCP*-containing-plasmid and pYY0040 were induced with IPTG to overproduce His-tagged ACPs/PCP. Twenty *E. coli* strains harboring both an *ACP/PCP*-containing-plasmid and a *PPTase*-containing-plasmid were induced with IPTG to overproduce His-tagged ACPs/PCP with intact PPTases. His-tagged ACPs/PCP were then purified to homogeneity by affinity chromatograph.

### Phosphopantetheinylation of the ACP/PCP involved in FK506 biosynthesis

**Figure 3:**
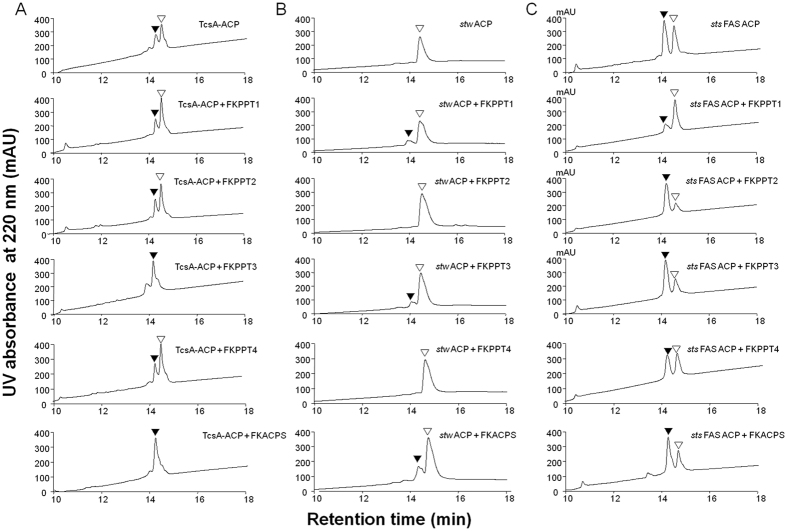
HPLC analyses of TcsA-ACP (**A**), *stw* ACP (**B**), and *sts* FAS ACP (**C**). Each of ACP genes was cloned into pET28a. Each of PPTase genes was cloned into pYY0040, a plasmid derived from pET44a. *E. coli* strains harboring both a *ACP*-containing-plasmid and a *PPTase*-containing-plasmid were induced with IPTG to produce His-tagged ACPs with intact PPTases. His-tagged ACPs were purified by affinity chromatograph and then analyzed by HPLC and MS. ▽, apo-form; ▼, holo-form.

Regarding TcsA-ACP, when *tcsA-ACP* was co-expressed with pYY0040 in *E. coli*, HPLC data showed that purified TcsA-ACP were eluted as two peaks with the area ratio of the small peak with shorter retention time and the large peak with longer retention time as 0.67. MS data revealed that the large peak represented apo-proteins and the small peak represented holo-proteins, indicating that *E. coli* ACPS could convert it from the apo-form to holo-form incompletely. After co-expression of *tcsA-ACP* with *FKPPT3* or *FKACPS*, all purified TcsA-ACP were holo-form by HPLC analysis. While co-expression of *tcsA-ACP* with *FKPPT1, FKPPT2* or *FKPPT4*, the ratio of the apo-form and the holo-form did not change compared with co-expression of it with pYY0040 (Fig. 3A). These results indicated that FKPPT3 and FKACPS could phosphopantetheinylate TcsA-ACP, but FKPPT1, FKPPT2, and FKPPT4 could not under these conditions.

**Figure 4:**
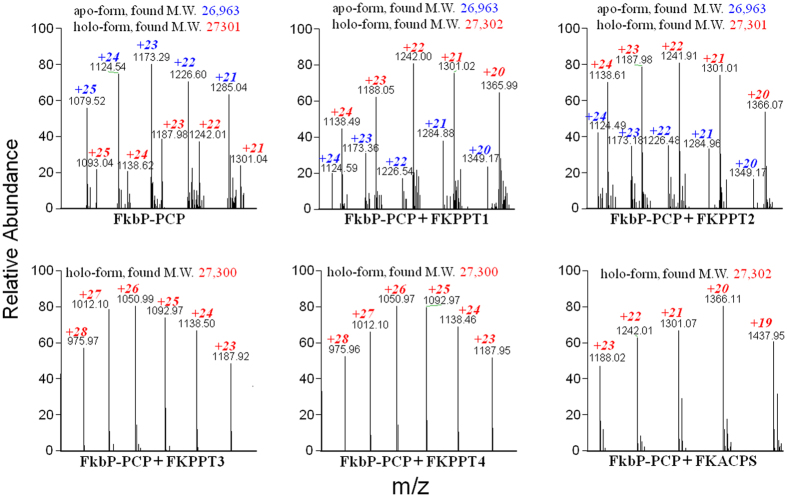
MS analyses of FkbP-PCP. FkbP-PCP gene fused with SUMO gene was cloned into pET28a. Each of PPTase genes was cloned into pYY0040. *E. coli* strains harboring both a *PCP*-containing-plasmid and a *PPTase*-containing-plasmid were induced with IPTG to produce His-tagged PCPs with intact PPTases. His-tagged PCPs were purified by affinity chromatograph and then analyzed by HPLC and MS. The calculated molecular weights of apo-form and holo-form of FkbP-PCP is 26,970 Da and 27,310 Da, respectively.

Regarding FkbP-PCP (fused with SUMO protein), when *FkbP-PCP* (fused with *SUMO* gene) was co-expressed with pYY0040 in *E. coli*, HPLC data showed that purified FkbP-PCP were eluted as a single peak. MS analysis showed that this peak represented both apo-proteins and holo-proteins with the ratio of the peak height of holo-proteins and the peak height of apo-proteins as about 0.40. After co-expression of *FkbP-PCP* with each of PPTase genes, HPLC data showed that purified FkbP-PCP were still eluted as a single peak. While co-expression of *FkbP-PCP* with *FKPPT1* and *FKPPT2*, MS data showed that the ratio of the peak height of holo-proteins and the peak height of apo-proteins increased significantly to 2.58 and 2.34, respectively. While co-expression of *FkbP-PCP* with *FKPPT3, FKPPT4*, and *FKACPS*, MS data showed that only the peak of holo-proteins remained but the peak of apo-proteins disappeared (Fig. 4). These results supported that all of these five PPTases could phosphopantetheinylate FkbP-PCP.

### Phosphopantetheinylation of the ACP in a type II PKS

When *stw ACP* was co-expressed with pYY0040 in *E. coli*, HPLC data showed that purified *stw* ACP were eluted as a single peak, which represented apo-proteins by MS analysis. After co-expression of *stw ACP* with *FKPPT1, FKPPT3*, or *FKACPS*, a new peak, whose area is about 21%, 20%, and 34% of that of apo-proteins respectively, with shorter retention time compared with that of apo-proteins appeared in HPLC data, which represented holo-proteins by MS analysis. After co-expression of *stw ACP* with *FKPPT2* or *FKPPT4*, HPLC data didn’t show any holo-proteins (Fig. 3B). These results suggested that *stw* ACP could be phosphopantetheinylated by FKPPT1, FKPPT3, and FKACPS but not FKPPT2 and FKPPT4.

### Phosphopantetheinylation of the ACP in FAS

When *sts FAS ACP* was co-expressed with pYY0040 in *E. coli*, HPLC data showed that purified *sts* FAS ACP were eluted as two peaks with the area ratio of the peak with shorter retention time and the peak with longer retention time as 1.20. MS data revealed that the peak with longer retention time represented apo-proteins and the peak with shorter retention time represented holo-proteins. After co-expression of *sts FAS ACP* with *FKPPT2, FKPPT3*, or *FKACPS*, the ratio of holo-proteins and apo-proteins increased significantly to about 2.93, 2.17, and 1.67, respectively. While co-expression of *sts FAS ACP* with *FKPPT4*, this ratio almost did not change (about 1.01). Surprisingly, while co-expression of *sts FAS ACP* with *FKPPT1*, this ratio decreased significantly to about 0.28 (Fig. 3C). These results supported that FKPPT2, FKPPT3, and FKACPS could phosphopantetheinylate *sts* FAS ACP, but FKPPT1 and FKPPT4 could not.

**Figure 5:**
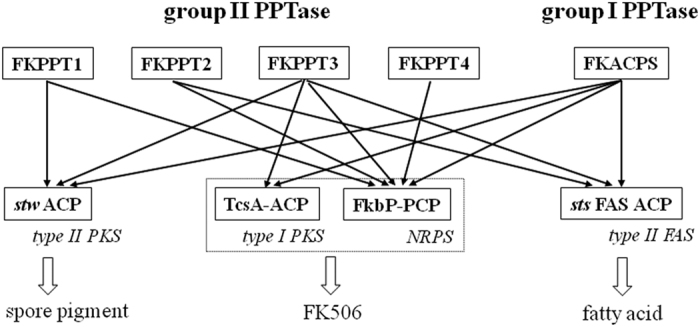
Proposed phosphopantetheinylation network in *S. tsukubaensis* L19.

The above results strongly supported that these five PPTases are indeed PPTases. Their functions should complement each other since each of ACPs/PCP could be phosphopantetheinylated by more than one PPTases, suggesting a complicate phosphopantetheinylation network in *S. tsukubaensis* L19 (Fig. 5). PCP could be phosphopantetheinylated by all five PPTases, suggesting that the flexibility to PPTases of PCP is more than that of ACPs.

### Inactivation of FKPPT1 or FKPPT3 decreased the FK506 yield in *S. tsukubaensis* L19

**Figure 6:**
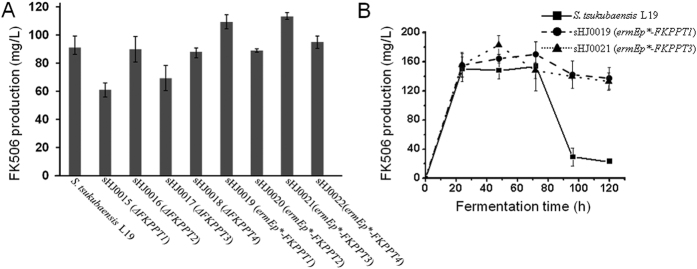
FK506 production of *S. tsukubaensis* L19 and its recombinant strains in flasks (**A**) and fermentors (**B**). A. Each strain was fermented in triplicate fermentation medium in flasks for 72 h. B. Each strain was fermented in triplicate fermentation medium in 100 L fermentors under rigorously identical conditions. sHJ0015, deletion of *FKPPT1* in *S. tsukubaensis* L19; sHJ0016; deletion of *FKPPT2* in *S. tsukubaensis* L19; sHJ0017, deletion of *FKPPT3* in *S. tsukubaensis* L19; sHJ0018, deletion of *FKPPT4* in *S. tsukubaensis* L19; sHJ0019, overexpression of *FKPPT1* in *S. tsukubaensis* L19; sHJ0020, overexpression of *FKPPT2* in *S. tsukubaensis* L19; sHJ0021, Overexpression of *FKPPT3* in *S. tsukubaensis* L19; sHJ0022, overexpression of *FKPPT4* in *S. tsukubaensis* L19.

To determine the influence of the activities of the group II PPTases on FK506 production *in vivo, FKPPT1, FKPPT2, FKPPT3*, and *FKPPT4* were individually replaced with the apramycin resistance gene *aac(3)IV* in *S. tsukubaensis* L19 by using Redirect technology, resulting in four mutant strains sHJ0015-sHJ0018, respectively. Each of mutant strains sHJ0015-sHJ0018 was fermented in triplicate fermentation medium in flasks for 72 h by using *S. tsukubaensis* L19 as control. FK506 production was monitored by HPLC analyses. The FK506 yields of sHJ0015 (*ΔFKPPT1*) and sHJ0017 (*ΔFKPPT3*) decreased ~33% and ~22% compared to that of *S. tsukubaensis* L19, respectively, suggesting that FKPPT1 and FKPPT3 play important roles in the FK506 biosynthesis. The FK506 yields of sHJ0016 (*ΔFKPPT2*) and sHJ0018 (*ΔFKPPT4*) had no obvious change compared to that of *S. tsukubaensis* L19, supporting that lack of FKPPT2 and FKPPT4 in the biosynthesis of FK506 could be complemented by other PPTases (Fig. 6A).

The above *in vivo* results showed that none of these four group II PPTases is indispensable to the FK506 biosynthesis. These results are consistent with the *in vitro* results that FK506 biosynthetic ACP and PCP could be phosphopantetheinylated by more than one PPTases.

### Overexpression of *FKPPT3* increased the FK506 production and decreased the fermentation time in *S. tsukubaensis* L19

To study the effect of the expression level of the group II PPTase genes on FK506 production, four PPTase gene overexpression mutant strains were constructed. *FKPPT1, FKPPT2, FKPPT3*, and *FKPPT4* under the control of a strong promoter ermEp* were individually introduced into *S. tsukubaensis* L19, resulting in four PPTase gene overexpression mutant strains sHJ0019-22, respectively. Each of mutant strains sHJ0019-sHJ0022 in triplicate cultures was fermented in flasks for 72 h by using *S. tsukubaensis* L19 as control. The FK506 yields of sHJ0020 (ermEp*-FKPPT2) and sHJ0022 (ermEp*-FKPPT4) had no obvious change compared to that of *S. tsukubaensis* L19, suggesting again that neither FKPPT2 nor FKPPT4 is crucial to the FK506 biosynthesis. However, the FK506 yields of sHJ0019 (ermEp*-FKPPT1) and sHJ0021 (ermEp*-FKPPT3) increased ~20% and ~25% compared to that of *S. tsukubaensis* L19, respectively, supporting again that FKPPT1 and FKPPT3 play important roles in FK506 biosynthesis (Fig. 6A). To confirm sHJ0019 (ermEp*-FKPPT1) and sHJ0021 (ermEp*-FKPPT3) enhance the abilities of FK506 production, these two strains were fermented in triplicate fermentation medium in 100 L fermentors under rigorously identical conditions by using *S. tsukubaensis* L19 as control. The curve of FK506 production in *S. tsukubaensis* L19 showed that the FK506 yield reached the highest level at 154 ± 34 mg/L at 72 h. Notably, the FK506 yields of sHJ0019 (ermEp*-FKPPT1) and sHJ0021 (ermEp*-FKPPT3) reached the highest level at 171 ± 17 mg/L at 72 h and 183 ± 13 mg/L at 48 h, respectively (Fig. 6B). Thus, sHJ0021 (ermEp*-FKPPT3) not only increased FK506 production by ~19% but also decreased the fermentation time by 24 h in the fermentor scale fermentation, which may be beneficial for the industrial FK506 production. Strain sHJ0021 was deposited in China General Microbiological Culture Collection Center (CGMCC) as name *Streptomyces tsukubaensis* L20 with CGMCC number 11252. Additionally, All of eight mutant strains didn’t show obvious morphological different comparing with *S. tsukubaensis* L19 during growth in solid media and liquid media, including sporulation, cell color, and the length of mycelium.

In summary, we identified that *S. tsukubaensis* L19 contains five discrete PPTases. Characterization of these PPTases showed that their functions complement each other, suggesting a complicate phosphopantetheinylation network in *S. tsukubaensis* L19. We also provided an example to improve the antibiotic production by engineering of PPTases.

## Methods

### Bacterial Strains, Plasmids, growth, and culture conditions

Bacterial strains and plasmids used in the present study are listed in Table 1 and primers are listed in Table 2. The spore preparation of *Streptomyces tsukubaensis* L19 was done on ISP4 agar after 10 days at 26 °C. DNA manipulations in *S. tsukubaensis* L19 and *E. coli*-*S. tsukubaensis* L19 conjugation were carried out according to standard procedures^33^.

### Fermentation of *S. tsukubaensis* L19 and its recombinant strains

*S. tsukubaensis* L19 and its recombinant strains were cultured on ISP4 agar at 26 °C for 7 days. Five full colonies were inoculated into 50 mL seed medium [1% (w/v) glycerol, 1% maltodextrin, 4% soybean meal, 0.2% CaCO_3_, pH 6.8] in 250 mL flasks and cultured at 28 °C, 220 rpm for 24–30 h. For fermentation of strains in flasks, the 2.5 mL of seed cultures were inoculated into 25 mL of fermentation medium (5% maltodextrin, 1% yeast extract, 3% cotton seed meal, 0.2% K_2_HPO_4_, 0.1% CaCO_3_, pH 6.8) and grown at 28 °C, 220 rpm for 5 days.

For fermentation of strains in fermentors, the 20 mL of seed cultures were inoculated into 10 L of seed medium containing 0.03% defoamer in 20 L fermentors and grown at 28 °C for 1 day. Then the 6 L of secondary seed cultures were inoculated into 60 L of fermentation medium containing 0.2% defoamer in 100 L fermentors and grown at 28 °C for 5 days. Fermentor cultivations were carried out by using the same bioreactor system with pH, pO_2_, and temperature control. All flask and fermentor experiments were performed at least triplicates.

### Analysis of FK506 production

To assay for FK506 production in the culture broths, a sample was withdrawn and ultrasonic extracted with the same volume of methanol. Methanol layer was recovered by centrifugation at 12,000 rpm for 15 min. The concentration of FK506 was determined using an HPLC system (Agilent Series 1100, Agilent) equipped with a SB-C18 column (150 × 2.1 mm, Agilent). The column temperature was maintained at 60 °C and UV detector was set at 215 nm. The mobile phase, which had a flow rate of 1.0 mL/min, contained 0.02 M KH_2_PO_4_ (pH 3.5) solution and acetonitrile in the ratio of 40:60.

### Genome sequencing and genome annotation

The nucleotide sequence of *S. tsukubaensis* L19 genome was determined by using a massively parallel pyrosequencing technology (Roche 454 GS FLX). 160 contigs (>500 bp) with a total size of 9.0 Mb were assembled from 522,882 reads (average length of 437 bp) using Newbler software of the 454-suite package, providing a 25.3-fold coverage. The relationship among contigs was determined by using multiplex PCR. Gaps were filled by sequencing PCR products. The final sequence assembly was performed using phred/phrap/consed package (http://www.phrap.org/phredphrapconsed.html), and the low sequence quality region was re-sequenced.

Putative protein-coding sequences were determined by combining the prediction results of glimmer 3.02^34^ and Z-Curve program^35^. Functional annotation of CDS was performed by searching the NCBI non-redundant protein database and KEGG protein database. Protein domain prediction and COG assignment were performed by RPS-BLAST using NCBI CDD library^36^.

### Production and purification of ACPs/PCP

The three ACP genes were amplified by PCR using relevant primers from genomic DNA of *S. tsukubaensis* L19. The resultant products were cloned into pTA2 vector (Toyobo) directly and sequenced to confirm PCR fidelity. Then these genes were digested with *Nde*I/*Hin*dIII and cloned into the same sites of pET28a (Novagen), yielding three *ACP*-containing-plasmids pET-AACP and pYY0081-pYY0082, respectively. And the PCP gene was cloned into the pET28a-SUMO (Novagen), resulting in a *PCP*-containing-plasmid pYY0098. Finally, these plasmids were introduced into *E. coli* BL21(DE3) to overproduce proteins as N-His_6_-tagged protein. The ACPs were overproduced under standard conditions. BL21(DE3) harboring each expression plasmid were grown in LB medium with kanamycin at 37 °C until OD_600_ reached 0.4. Then IPTG was added to the final concentration of 0.4 mM and incubation continued at 37 °C for 4 h, resulting in overproduction of proteins in soluble form with good yield.

Purification of these proteins by affinity chromatography on Ni-NTA agarose (Qiagen) was performed under standard conditions recommended by the manufacturer. The proteins were dialyzed against 20 mM Tris·HCl (pH 8.0), 25 mM NaCl, 1 mM DTT, and 10% glycerol.

### Co-expression of ACP/PCP genes with PPTase genes

The five PPTase genes were amplified by PCR using relevant primers from genomic DNA of *S. tsukubaensis* L19. The resultant products were cloned into pTA2 vector directly and sequenced to confirm PCR fidelity. Then these genes were digested with *Nde*I/*Hin*dIII and cloned into the same sites of pET44a (Novagen), yielding five *PPTase*-containing-plasmids pYY0072-pYY0074 and pYY0077-pYY0078, respectively. BL21(DE3) harboring both each of *ACP/PCP*-containing-plasmids and each of *PPTase*-containing-plasmids were grown in LB medium with both kanamycin and ampicillin at 37 °C until OD_600_ reached 0.4. Then IPTG was added to the final concentration of 0.4 mM and incubation continued at 37 °C for 4 h. Purification and dialysis of ACPs/PCP was performed as same as described before.

### HPLC analyses of ACPs

The ACPs produced from *E. coli* or from the phosphopantetheinylation reaction mixture were directly analyzed by HPLC (LC-20AT, Shimadzu). HPLC separation was carried out on a C18 column (Zorbax, 300SB-C18, 5 μm, 4.6 × 250 mm, Agilent). Solvent A consisted of 0.1% formic acid. Solvent B consisted of acetonitrile. The following binary gradient was used: 0–15 min, a linear gradient from 0% to 75% B; 15–16 min, a linear gradient from 75% to 100% B; 16–19 min, 100% B; 19–20 min, a linear gradient from 100% to 0% B; 20–24 min, 0% B at a flow rate of 1 ml/min. UV detection was performed at 220 nm (SPD-20A, Shimadzu).

### LC-MS analyses of ACPs/PCP

The ACPs produced from *E. coli* or from the phosphopantetheinylation reaction mixture were directly analyzed by LC-MS (Agilent 1200, Thermo Finnigan LCQ Deca XP MAX). LC separation was carried out on a Agilent SB-C18 column (3.5 μm, 80 Å, 2.1 × 150 mm, Agilent) at 35 °C. Solvent A consisted of 0.1% formic acid. Solvent B consisted of acetonitrile. The following binary gradient was used: 0–5 min, 5% B; 5–45 min, a linear gradient to 75% B; followed by 3 min isocratic elution of 75% B, and equilibrated to initial condition for 13 min at a flow rate of 0.2 ml/min. UV detection was performed at both 254 nm and 280 nm. MS equipped with ESI source was performed as follows: positive; source voltage, 2.5 kV; capillary voltage, 41 V; sheath gas flow, 45 arb; aux/sweep gas flow, 5 arb; capillary temperature, 330 °C.

### Construction of *PPTase:aac3(IV)* mutant strains

The PPTase gene disruption mutant was constructed by using a modified PCR targeting system as follows^37^. First, four cosmids, which contain each of *FKPPT1, FKPPT2, FKPPT3*, and *FKPPT4*, were screened out by PCR amplification. Second, each disruption cassette *aac(3)IV* (Apra) was PCR amplified from pHY773 (Z. Qin, Institute of Plant Physiology and Ecology, Chinese Academy of Sciences, Shanghai, China, unpublished results), with the resulting product carrying 59 bp ends with homology to the corresponding region of PPTase gene. Each PCR product was then introduced into *E. coli* BW25113 carrying pIJ790/cosmid, and transformed cells carrying mutagenized cosmid were selected on LB agar containing apramycin. Each mutagenized cosmid, in which a PPTase gene was replaced with *aac(3)IV*, was confirmed by PCR analysis using primers accordingly. Third, after conjugal transfer of each mutagenized cosmid from *E. coli* ET12567 carrying pUZ8002 into *S. tsukubaensis* L19, exconjugants were obtained after selection for apramycin resistance. Exconjugants were then inoculated onto ISP4 plates for two rounds of nonselective growth before selection by replica plating for thiostrepton-sensitive and apramycin-resistant colonies. Each resulting strain, in which a PPTase gene was replaced with *aac (3)IV*, was confirmed by PCR analysis using primers accordingly. The disruption of *FKPPT1, FKPPT2, FKPPT3* and *FKPPT4* in *S. tsukubaensis* L19 were performed as described above, resulting in mutant strains sHJ0015-sHJ0018, respectively.

### Construction of PPTase gene overexpression mutant strains

A site-specific integration vector pIJ8660^38^, containing *egfp, Ф31 int* and *attP*, was used to construct an integration recombinant plasmid. The *egfp* was replaced with ermEp* promoter and a multiple cloning site. The *Nde*I/*Not*I DNA fragments of each PPTase gene were cloned from *S. tsukubaensis* L19 into the same sites of pIJ8660, resulting in plasmids pYY0099-pYY0102. Each of pYY0099-pYY0102 was transferred to *S. tsukubaensis* L19 via conjugal transfer from *E. coli* ET12567 (pUZ8002) using standard procedures. The resulting strains, in each of which a PPTase gene was expressed under the control of ermEp*, were designated as strains sHJ0019-sHJ0022 and confirmed by PCR analysis.

## Additional Information

**How to cite this article**: Wang, Y.-Y. *et al*. Characterization of Discrete Phosphopantetheinyl Transferases in *Streptomyces tsukubaensis* L19 Unveils a Complicate Phosphopantetheinylation Network. *Sci. Rep.* **6**, 24255; doi: 10.1038/srep24255 (2016).

## Figures and Tables

**Table 1:** List of plasmids and strains used in this study.

	Description	Reference
Plasmids		
pYY0040 pET-AACP	Deletion of both His-Tag gene and Nus-Tag gene from pET44a *tcsA-ACP* cloned as *Nde*I/*Hin*dIII fragment into pET28a	16,28
pYY0081	*stw ACP* cloned as *Nde*I/*Hin*dIII fragment into pET28a	This study
pYY0082	*sts FAS ACP* cloned as *Nde*I/*Hin*dIII fragment into pET28a	This study
pYY0098	*fkbP-PCP* cloned as *Nde*I/*Hin*dIII fragment into pET28a	This study
pYY0072	*FKPPT1* cloned as a *Nde*I/*Hin*dIII fragment into pET44a	This study
pYY0078	*FKPPT2* cloned as a *Nde*I/*Hin*dIII fragment into pET44a	This study
pYY0073	*FKPPT3* cloned as a *Nde*I/*Hin*dIII fragment into pET44a	This study
pYY0077	*FKPPT4* cloned as a *Nde*I/*Hin*dIII fragment into pET44a	This study
pYY0074	*FKACPS* cloned as a *Nde*I/*Hin*dIII fragment into pET44a	This study
pYY0099	*FKPPT1* cloned as a *Nde*I/*Not*I fragment into pIJ8660	This study
pYY0100	*FKPPT2* cloned as a *Nde*I/*Not*I fragment into pIJ8660	This study
pYY0101	*FKPPT3* cloned as a *Nde*I/*Not*I fragment into pIJ8660	This study
pYY0102	*FKPPT4* cloned as a *Nde*I/*Not*I fragment into pIJ8660	This study
Strains		
* S. tsukubaensis* L19	An industrial FK506 producing strain	This study
sHJ0015	Deletion of *FKPPT1* in *S. tsukubaensis* L19	This study
sHJ0016	Deletion of *FKPPT2* in *S. tsukubaensis* L19	This study
sHJ0017	Deletion of *FKPPT3* in *S. tsukubaensis* L19	This study
sHJ0018	Deletion of *FKPPT4* in *S. tsukubaensis* L19	This study
sHJ0019	Overexpression of *FKPPT1* in *S. tsukubaensis* L19	This study
sHJ0020	Overexpression of *FKPPT2* in *S. tsukubaensis* L19	This study
sHJ0021	Overexpression of *FKPPT3* in *S. tsukubaensis* L19	This study
sHJ0022	Overexpression of *FKPPT4* in *S. tsukubaensis* L19	This study

**Table 2:** List of primers used in this study.

Primer	Sequence	Note
WYY31	GCGCAAGCTTtcagtctccgtcgaggtcgg	For expression of *FKPPT1*
WYY32	GCGCCATATGgccacgaccgccgagca	
FK188	TACAACCGAAGAACCGCAGAAA	For screening *FKPPT1*
FK189	GGCTATGACCGTGAGCAGGA	
FK190	gccgtacaaccgaagaaccgcagaaaagacgaggtttacATTCCGGGGATCCGTCGACC	For *FKPPT1:aac(3)IV* mutant
FK191	cagccccggaagcgtcatgaaaatgatgaactgcatgcgTGTAGGCTGGAGCTGCTTC	
WYY39	GCGCAAGCTTtcattgggccacccccaccg	For expression of *FKPPT2*
WYY40	GCGCCATATGagcgcggctgtccggcc	
FK196	TCGGCGATCTGCATCTGCT	For screening *FKPPT2*
FK197	TACCCCTTCCGCCTTCTCC	
FK198	ggtcttcagcgcgcgggcgctgaccgggacggccgggtcATTCCGGGGATCCGTCGACC	For *FKPPT2:aac(3)IV* mutant
FK199	cgggtgccccggagacccgtaccccttccgccttctccgTGTAGGCTGGAGCTGCTTC	
WYY45	GCGCCATATGgaggacgatcggccggg	For expression of *FKPPT3*
WYY46	GCGCAAGCTTtcaggctccggttcggtgcc	
FK177	tcgccgacgacgcatgaacgt	For screening *FKPPT3*
FK178	ACATTCGGCTCGGTCGGGCTCA	
FK179	cacccgtggcggcgctgcttaccgtgctggagattgcccATTCCGGGGATCCGTCGACC	For *FKPPT2:aac(3)IV* mutant
FK180	gtcgagggattccggcgccgtccgccggggacggacgaTGTAGGCTGGAGCTGCTTC	
WYY51	GCGCAAGCTTtcagcggtcgtccggtggat	For expression of *FKPPT4*
WYY52	GCGCCATATGgcagcgctgaccaccgg	
FK204	CCGTACCTGACCGTCCCTC	For screening *FKPPT4*
FK205	GCACTCGTTGGCGTCCTCC	
FK206	ccgcggggcgggaggccccatgcgaacaggaggcaccccATTCCGGGGATCCGTCGACC	For *FKPPT4:aac(3)IV* mutant
FK207	aactgcgaccggcaccggccggaaccggtcgctccggggTGTAGGCTGGAGCTGCTTC	
WYY37	GCGCCATATGatcatcggggtggggat	For expression of *FKACPS*
WYY38	GCGCAAGCTTctacccctccgcgatcacca	
FK159	GCGCAGATCTATGctgccccggaccacgagc	For expression of *FKbP-PCP*
FK160	GCGCTCTAGAgggtgcgggccccgcacc	
FK119	GCGCCATatggccgccacgcaggaaga	For expression of *sts FAS ACP*
FK120	GCGCAAGCTTtcagccctggtggtcgagga	
FK121	GCGCCATatggacagcctgacctccgg	For expression of *stw ACP*
FK122	GCGCAAGCTTtcaggctccgctcttgagaa	
Pri53	TCCTAAGGATCCGGCGGCTTGCGCCCGATGCTAGTC	For ermEp*
